# Multi-Detector Helical Computed Tomography, Transrectal Ultrasonography, and Histology of the Sacroiliac Joint: A Comparative Study in Adult Warmblood Horse Cadavers

**DOI:** 10.3390/ani15131995

**Published:** 2025-07-07

**Authors:** Rebecca A. Mathys, Thomas R. Schmitz, Hans Geyer, Nicole Borel, Monika Hilbe, Stefanie Ohlerth, Andrea S. Bischofberger

**Affiliations:** 1 Equine Department, Vetsuisse Faculty, University of Zurich, 8057 Zurich, Switzerland; 2 Institute of Veterinary Anatomy, Vetsuisse Faculty, University of Zurich, 8057 Zurich, Switzerland; 3 Institute of Veterinary Pathology, Vetsuisse Faculty, University of Zurich, 8057 Zurich, Switzerland; 4 Clinic for Diagnostic Imaging, Vetsuisse Faculty, University of Zurich, 8057 Zurich, Switzerland

**Keywords:** equine, sacroiliac joint disease, anatomy, histology, computed tomography, transrectal ultrasonography

## Abstract

This study described, assessed and correlated ultrasonographic, computed tomographic, and histological findings in the sacroiliac joints of adult Warmblood horse cadavers. In total, 25 joints from 15 horses were examined post-mortem using transrectal ultrasonography and helical computed tomography. Findings on computed tomography were graded in the caudal joint third (caudally) and for the entire joint as mild, moderate, or severe. In total, 11 joints from nine horses were evaluated histologically. All joints (100%) showed abnormalities on computed tomography, and 92% (23/25) displayed abnormal ultrasonographic findings. The most common ultrasonographic findings were osteophytes (92%), joint effusion (76%), and sacral/iliac bone modeling (76%). Computed tomography revealed osteophytes (92% caudally, 100% overall), sclerosis (72% caudally, 88% overall), subchondral bone lesions (60% caudally, 88% overall), and enthesophytes (60% caudally, 68% overall). The most severe CT findings occurred caudally (44%), whereas 24% occurred cranially,16% in the mid portion of the SIJ, and 16% were multifocally present in the SIJ Histological analysis showed degenerative changes in the cartilage, subchondral bone, and the joint capsule. Horses with more pronounced imaging abnormalities also showed corresponding histological degeneration. Significant correlations were found between computed tomographic findings caudally and in the entire joint (r_s_ = 0.915, *p* < 0.001, *n* = 25), and between imaging and histological findings (computed tomography: r_s_ = 0.731, *p* = 0.011, *n* = 11; ultrasonography: r_s_ = 0.67, *p* = 0.024, *n* = 11). Non-significant correlations were observed between mean ultrasonographic and computed-tomographic grades (r_s_ = 0.35, *p* = 0.087, *n* = 25). Findings suggest these structural changes may reflect adaptation to joint loading. Transrectal ultrasonography appears suitable as a first-line diagnostic tool. However, future studies are essential to compare the diagnostic imaging findings of SIJs in asymptomatic and symptomatic horses to elucidate their clinical relevance.

## Simple Summary

Sacroiliac joint (SIJ) disease is a common cause of poor performance in horses, but diagnosis is challenging due to the joint’s deep anatomical location. A common diagnostic method is transrectal ultrasound and nuclear scintigraphy. Newer techniques, such as computed tomography, may provide a more complete evaluation, but their diagnostic value in assessing the sacroiliac joint in horses is still unclear. This study evaluated 25 sacroiliac joints of 15 adult Warmblood horses without reported pain using post-mortem transrectal ultrasonography, computed tomography, and histological examination. All joints showed structural changes on computed tomography, and most also presented abnormalities on ultrasonography. The most severe changes were observed in the caudal third of the joint, which is the region accessible by transrectal ultrasonography. Histological analysis revealed degenerative changes in the articular cartilage, subchondral bone, and joint capsule. Horses with more pronounced imaging abnormalities also showed corresponding degenerative changes on histology. The results indicate that structural changes in the sacroiliac joint are common in owner-perceived sound horses and may be part of normal ageing or adaptation to training. Future studies are essential to compare the diagnostic imaging findings of SIJs in asymptomatic and symptomatic horses, to elucidate the clinical relevance of these findings.

## 1. Introduction

Sacroiliac joint (SIJ) disease is a common cause of poor performance in sport horses [1,2,3]. Dressage and show jumping horses in particular are at a higher risk of developing SIJ disease than horses in other disciplines [1]. Diagnosing SIJ pain can be challenging [4]. A thorough orthopedic examination including SIJ anesthesia is key to rule out other conditions resulting in similar clinical presentations, such as thoracolumbar pain, lumbosacral pain, and mild hind limb lameness of other origins, such as proximal suspensory desmopathy or mild ataxia [1,2,3].

Diagnostic imaging of the SIJ and surrounding structures includes nuclear scintigraphy or transrectal ultrasonography [5,6,7,8,9,10]. The sensitivity and specificity of nuclear scintigraphy for diagnosing SIJ pain were found to be 51% and 77%, respectively [11]. The ultrasonographic evaluation protocol of the normal SIJ, staging of disease severity, and its imaging features have previously been reported [10,12,13,14,15,16]. Albeit being fast and cheap, evaluation of the SIJ via transrectal ultrasonography is limited to the caudoventral aspect of the joint, raising the question of whether pathologic changes in the dorsolateral aspect of the joint remain underdiagnosed [15]. So far, there is only limited information available on the correlation of ultrasonographic changes of the SIJ with other diagnostic imaging modalities.

In human medicine, computed tomography (CT) is a frequently used imaging modality for patients experiencing pain in the lumbosacroiliac region [17]. Recent studies in dogs have established CT as a sensitive modality to detect degenerative SIJ [18,19,20]. Good anatomical detail was shown when the equine lumbosacral region was examined with CT in living patients and cadavers [13,21,22]. With the development of newer, large-bore CT units, examination of the SIJ in horses under general anesthesia is becoming a feasible diagnostic option.

The equine SIJ is a tight joint (amphiarthrosis) and lies at an approximately 30° angle from the horizontal plane between the first sacral transverse process and the wing of the ilium [23]. The main functions of the SIJ are the fixation of the sacrum between the wing of the ilium and the transmission of propulsive forces from the hind limbs to the trunk [24]. The SIJ has been described as an atypical synovial joint because the sacral joint surface appears to be covered with hyaline cartilage, whereas the iliac joint surface is covered with fibrocartilage [4,23,25,26]. The joint is enclosed by a joint capsule closely adjacent to the articular surface and is supported by three sacroiliac ligaments [4,27]. When horses show chronic poor performance and pain that has been localized to the lumbosacroiliac region using infiltration with local anesthetic solution, distinct histological abnormalities in nerve sections and macroscopic osseous variations in the sacral and lumbar regions can be observed [28]. These findings highlight the importance of correlating clinical signs with the underlying histopathological changes associated with SIJ disease in horses.

Currently, there are no comparative reports between the CT changes of the equine SIJ with other diagnostic modalities, such as transrectal ultrasonography and histology. The objectives of this study were to describe, grade, and correlate transrectal ultrasonographic, CT, and histological findings of the SIJs in Warmblood horse cadavers. We hypothesized that SIJ changes are common in Warmblood horses without reported clinical signs of SIJ disease before euthanasia, and that sacroiliac joint changes detected by computed tomography (CT) would exhibit a strong correlation with findings obtained through transrectal ultrasonography. Additionally, we postulated that horses exhibiting SIJ alterations detectable through imaging modalities (CT and/or ultrasonography) would also present corresponding histopathological changes.

## 2. Materials and Methods

Warmblood horses aged 5–18 years (mean age: 12.3 ± 3.81 years) that were euthanized or slaughtered for reasons unrelated to the study were included in the investigation following owners’ consent at the Equine Hospital, University of Zurich between September 2021 and May 2023. All horses had been regularly used as riding horses in their respective disciplines and, according to the owner or trainers, exhibited no clinical signs indicative of SIJ disease.

### 2.1. Diagnostic Imaging

**Figure 1:**
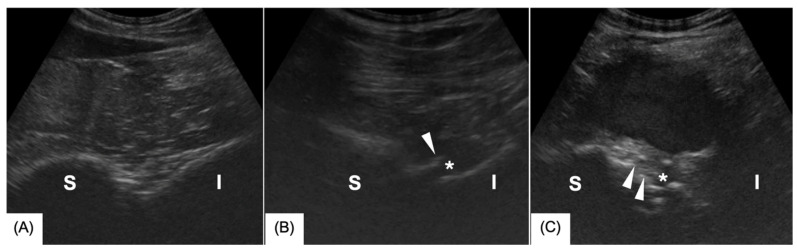
Transrectal ultrasound images of the sacroiliac joint including the sacrum (S) and the ilium (I): (**A**) Mild changes (Grade 1) consisting of mild osteophyte formation and mild joint effusion. (**B**) Moderate changes (Grade 2) consisting of moderate joint effusion (asterisk) and noticeable osteophyte formation (arrowhead). (**C**) Severe changes (Grade 3) including marked modeling of the sacrum and ilium, pronounced joint effusion, and extensive osteophyte formation.

Immediately after euthanasia or slaughter, a transrectal ultrasonographic examination was performed using a curvilinear probe (Hitachi-Aloka ProSound Alpha 7, Hitachi Healthcare, Tokyo, Japan) with the horse positioned in left lateral recumbency. The ultrasonographic examination was conducted by an ECVS diplomate in training as a large animal diagnostic imaging resident (3rd year) or an ECVDI-certified radiologist and adhered to a previously published protocol [12]. The images were graded according to Tallaj et al. [10]. Briefly, the following criteria were evaluated in all joints (*n* = 25): articular margins (osteophyte formation), modeling (shape change) of the sacrum and ilium, ventral sacroiliac ligament changes, enthesophyte formation, and joint effusion (see Table 1). Findings were graded on a scale from 0 to 3, whereby 0 represented no abnormal findings, 1 represented mild, 2 represented moderate, and 3 represented severe findings (Figure 1A–C). In the end, an overall mean score was given for each joint. Grade 0 was given for normal joints, and Grade 1 was assigned if one or more criteria were mild. If at least one criterion was moderate, Grade 2 was given. Grade 3 was assigned if one or more criteria were graded as severe.

Within one to four hours following death, the abdominal and pelvic organs were removed. The pelvis was isolated cranially by disarticulation between the 3rd and 4th lumbar vertebrae, caudally by osteotomy at the first caudal vertebra, and distally in the mid-femur region. To facilitate specimen handling and reduce CT-artifacts, such as streaking and image noise, the pelvis was bisected through the symphysis, the midline of the sacrum, and the vertebral canal using a bone saw. This approach further enabled the precise positioning of each sacroiliac joint within the isocenter of the CT gantry. The specimens were subsequently frozen at −28 °C until diagnostic imaging was performed. Before CT imaging, they were slowly thawed in warm water for 24–48 h.

All CT images were obtained using a multidetector 160-slice (dual) 80-slice scanner (Canon Aquilion Exceed LB, Canon Medical Systems AG Corporation, Wallisellen, Switzerland.) in a helical acquisition mode, with a pitch of 35.9 (pitch factor 0.898), 135 kV, 530 mAs, a field of view of 700 mm, and a matrix of 256 × 256. Images were reconstructed in a bone and soft tissue algorithm with a slice thickness of 1 mm.

An ECVDI-certified radiologist (S.O.) and an ECVS diplomate training as a large animal diagnostic imaging resident (3rd year) (A.B.) reviewed all CT images. The SIJ length was measured once in the frontal plane at its maximal extent. This measurement was divided into three thirds. The caudal third of the SIJ as well as the complete SIJ were then evaluated and graded separately. The grading was adapted from Wise et al. [20]. Briefly, the CT criteria included osteophyte formation, periarticular modeling (enthesophyte formation), subchondral bone sclerosis, subchondral bone lesions, and sacrum/ilium modeling (shape change). Each criterion was assigned a severity grade: 0 = none, 1 = mild, 2 = moderate, and 3 = severe (Table 2). An overall mean score was calculated for the caudal third and the complete SIJ. Additionally, the most severe findings in each joint were localized to either the caudal, middle, or cranial third of the joint.

### 2.2. Histological Examination

Before the main study, sampling and histological processing were tested on a pilot cadaver. SIJ histology was performed on 11 randomly chosen SIJs following the CT examination. All muscles were removed from the pelvic halves. The SIJ, together with the lumbosacral and intertransverse joints, was sectioned into three blocks, each measuring 10 cm in length, 4 cm in width, and 6 cm in height, using a bone saw. The blocks were fixed in a buffered solution containing 2.5% glutaraldehyde and 1.3% formaldehyde (Fluka Chemie GmbH, Buchs, Switzerland). The fixation process lasted one week, during which a shaker was used to enhance the infiltration of the fixative. The fixed blocks were dehydrated in ethanol (Fluka Chemie GmbH, Buchs, Switzerland) and subsequently infiltrated and embedded in methyl methacrylate (MMA) (Brunschwig AG, Supplies for Laboratory and Production, Basel, Switzerland). The entire infiltration and embedding process required 12 weeks to complete. The MMA blocks were sectioned sagittally every 2 mm using a diamond-coated band saw (Haslab GmbH, Labor und Medizintechnik, Ostermundigen, Switzerland). The resulting cuts typically measured 1.2 mm in thickness and were subsequently bonded to a polyacrylic plate. The sections were ground to a thickness of 300–500 μm using a Leica SP 2600 milling machine (Leica Microsystems Schweiz AG, Glattbrugg, Switzerland). Staining was performed using either Giemsa or van Kossa/McNeal techniques. The van Kossa/McNeal staining technique was utilized to examine the subchondral bone and to detect calcifications in the articular cartilage associated with degenerative changes. Four authors (H.G., N.B., M.H., and R.M.) examined the stained slides with a Leica stereomicroscope, a Leica DMR microscope, and a Leica IM 1000 image manager (Leica Microsystems Schweiz AG, Glattbrugg, Switzerland). The sections were examined for the presence and distribution of hyaline cartilage and fibrous cartilage in the sacroiliac joint. The thickness of the cartilage and subchondral bone was measured using an image manager (Leica Microsystems Schweiz AG, Glattbrugg, Switzerland). The maximum thickness, minimum thickness, and corresponding mean thickness values were determined. In addition, the presence of degenerative changes within the articular cartilage, subchondral bone plate, and joint capsule of the SIJ were thoroughly assessed. These changes were graded according to a grading system modified by Mcllwraith et al. [29] (Table 3). Each histological section of the respective joint was graded individually, with the sacral and iliac joint surfaces assessed separately. A mean score for each joint was then calculated by averaging the scores of all its histological sections. The histological changes were then categorized as mild (score 1–9/26), moderate (score 10–18/26), or severe (score 19–26/26).

### 2.3. Statistical Analysis

Data were stored in Microsoft Excel program (Microsoft Excel for Mac 2024, version 16.87, Redmond, WA, USA). All statistical analyses were performed with SPSS 29 statistical software (IBM SPSS Statistics, Chicago, IL, USA). Data distribution was tested with a Kolmogorov–Smirnov test. A *p*-value of <0.05 was considered statistically significant. The Spearman’s rank correlation coefficient was calculated to evaluate the correlation between the overall scores of the ultrasonographic and CT findings, and between imaging modalities and histology. A paired *t*-test was used to compare the subchondral bone thickness and the cartilage thickness between the ilium and sacrum.

## 3. Results

A total of 15 Warmblood horses were included for imaging examination. In total, 25 sacroiliac joints (SIJ) provided images of sufficient quality for evaluation. Reasons for euthanasia or slaughter included blindness (*n* = 1), acute severe neurological signs (*n* = 2), severe acute laminitis (*n* = 3), behavioral issues (*n* = 2), colic (*n* = 4), headshaking (*n* = 2), and acute superficial flexor tendon rupture (*n* = 1). The horses had been used for show jumping (*n* = 7), dressage (*n* = 2), or pleasure riding (*n* = 6). Histological examination was performed on 11 randomly chosen SIJs.

### 3.1. Ultrasonography

The ultrasonographic examination revealed abnormal findings in 92% of the sacroiliac joints. Among these, 40% exhibited mild findings, 44% displayed moderate findings, and 8% were classified as severe findings (Figure 1A–C). The most common ultrasonographic findings were osteophytes (92%), effusion (76%), and sacral/iliac bone modeling (76%). Enthesophytes were observed in 68% of the cases, while alterations in the ventral sacroiliac ligament were detected in 52%.

### 3.2. CT

All (100%) SIJs exhibited abnormal findings on CT examination. Consistent with ultrasonographic results, 44% of the joints showed mild changes, 48% demonstrated moderate changes, and 8% were classified as having severe changes. The most prevalent CT findings in the SIJ included osteophytes (92% in the caudal third and 100% in the entire SIJ), sclerosis (72% and 88%, respectively), subchondral bone lesions (60% and 88%, respectively), and enthesophytes (60% and 68%, respectively). Among the CT scans, the most severe changes were located in the caudal third of the joint in 44% of cases (11/25), in the middle third in 16% (4/25) and the cranial third in 24% (6/25). In the remaining SIJs (4/25, 16%), the most severe lesions could not be assigned to a specific area within the SIJ. While reviewing the CT images, we largely found two different types of changes: either primary subchondral bone changes, such as subchondral cyst-like lesions with associated subchondral bone thickening, and sclerosis or periarticular new bone formation bridging the cranial and caudal aspects of the SIJ (referred to as ventral buttressing).

### 3.3. Histology

The sagittal thick sections obtained in this study provided an excellent depiction of the anatomy of the equine SIJ. In the sections involving the SIJ, the joint with the sacral and iliac joint surfaces and the corresponding subchondral bone plates, the joint capsule (especially at the caudal joint margin), and often parts of the ventral and interosseous sacroiliac ligaments were histologically assessable.

**Figure 2:**
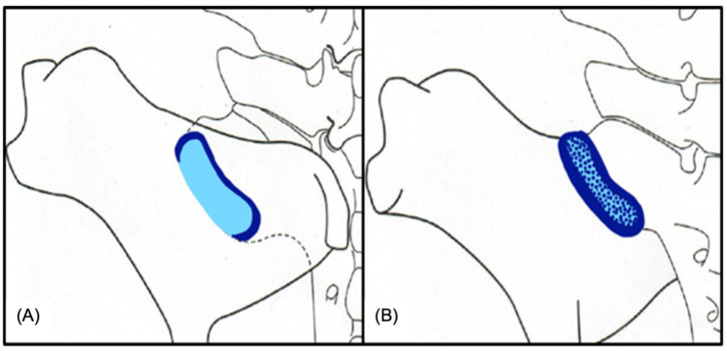
Illustration of cartilage type distribution within the SIJ. (**A**) dorsal view of the sacral articular surface, depicting the distribution of cartilage types. The central and caudal regions of the joint are covered by hyaline cartilage (light blue); the cranial, lateral, and medial joint margins predominantly by fibrocartilage (dark blue). (**B**) ventral view of the iliac articular surface. The cranial, caudal, lateral and medial joint margins are predominantly covered by fibrocartilage (dark blue). The central portion of the joint typically consists of a mixture of hyaline and fibrocartilage (a mixture of dark- and light-blue areas).

**Figure 3:**
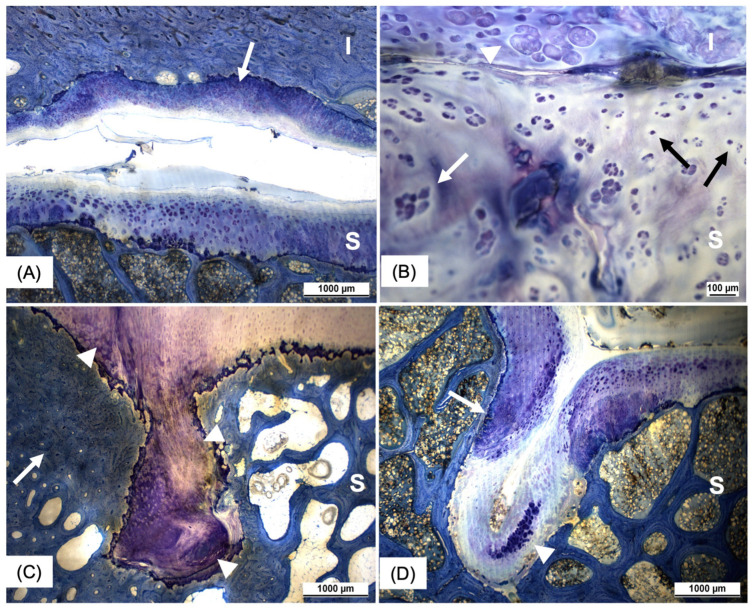
(**A**–**D**). Sagittal histological sections of the SIJ stained with Giemsa stain. (**A**) histological section with low magnification shows the presence of hyaline cartilage in both the sacral (S) and iliac (I) articular cartilage. The arrow depicts the transition between hyaline and fibrocartilage in the iliac articular cartilage, which was frequently seen in the center of the iliac articular cartilage. (**B**) a histological section in middle magnification through the iliac (I) and sacral (S) articular surface showing several of the commonly observed histological signs of articular cartilage changes. The white arrow shows a clustering of chondrocytes without concurrent hypertrophy. The arrowhead depicts clustering of chondrocytes with hypertrophy, and the black arrows show pyknotic nuclei visible within the iliac articular cartilage. (**C**) histological section at the sacral (S) joint surface in low magnification, showing a subchondral cyst-like lesion in the subchondral bone. The white arrow marks the associated subchondral bone sclerosis. The arrowheads indicate a focal replacement of the hyaline cartilage normally present at this site with fibrocartilage. (**D**) sagittal histological section stained with Giemsa stain in low magnification. Indentation within the sacral (S) subchondral bone with focal replacement of hyaline cartilage with fibrocartilage (arrow) and clustering of chondrocytes without hypertrophy (arrowhead).

In terms of cartilage distribution, the iliac articular surface was peripherally composed of fibrocartilage, while a combination of hyaline and fibrocartilage was evident in the center of the joint surface (Figure 2 and Figure 3A). The sacral articular surface was covered by fibrocartilage at the cranial, lateral, and medial joint margins, whereas the caudal margin and the center were covered by hyaline cartilage. The mean cartilage thickness on the sacrum (1.19 ± 0.29 mm) was significantly thicker than on the ilium (0.86 ± 0.19 mm), (*p* < 0.001). In contrast to that, the subchondral bone of the ilium (2.74 ± 0.85 mm) was significantly thicker compared to the subchondral bone of the sacrum (0.51 ± 0.22 mm), (*p* < 0.001) (Figure 3A). The most frequently observed histological findings are summarized in Table 4. The occurrence of chondrocyte group formation, both with and without hypertrophy (Figure 3B,D), hypertrophy in the absence of group formation, tidemark perforation, pyknotic chondrocytes (Figure 3B), and subchondral bone sclerosis (Figure 3C) was significantly more prevalent in the sacral joint surface compared to in the iliac joint surface. When all histological criteria applied were summarized in a total score, the majority of SIJ (90.9%) had mild changes, 9.1% of SIJ had moderate changes, and none of the SIJ showed severe changes.

### 3.4. Correlation Between Imaging Modalities (CT, US) and Histology

A non-significant correlation was found when comparing the mean overall ultrasonographic grade with the CT grade of the entire SIJ (*p* = 0.087, correlation coefficient r_s_: 0.35, *n* = 25) or the caudal part of the SIJ (*p* = 0.15, correlation coefficient r_s_: 0.29, *n* = 25). The CT changes in the caudal third of the SIJ strongly and significantly correlated with the changes of the entire SIJ (*p* < 0.001, correlation coefficient = 0.918, *n* = 25). When enthesophytes were evaluated separately, a high correlation between the ultrasonography and CT was detected (*p* < 0.001; correlation coefficient r_s_: 0.728, *n* = 25).

**Figure 4:**
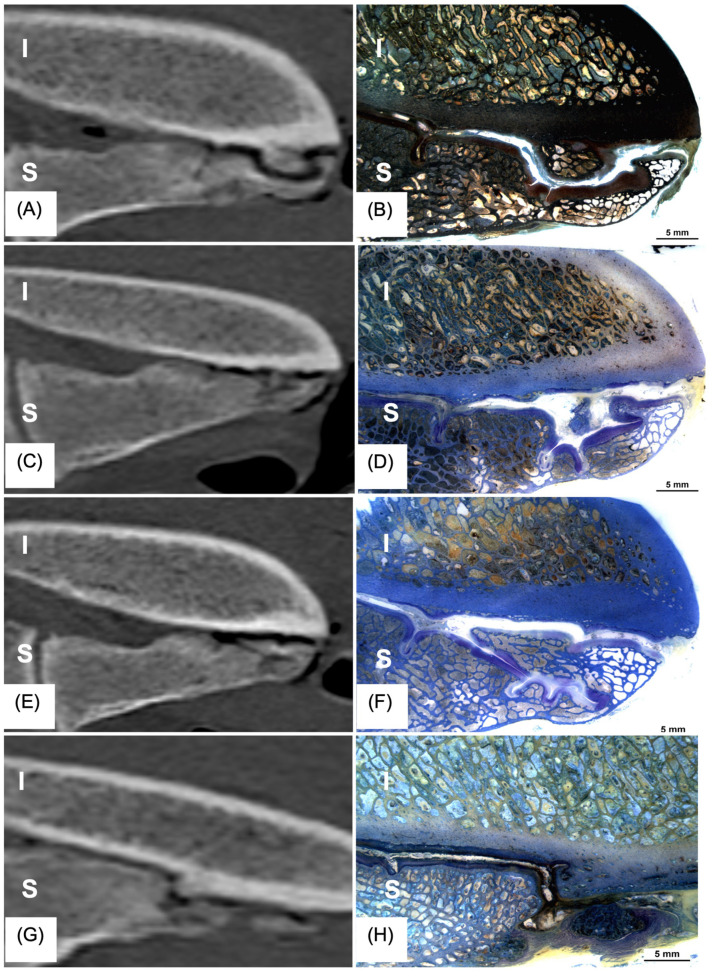
(**A**–**H**). Illustration comparison of sagittal helical CT- and histological sections of the equine SIJ showing typical imaging features and corresponding histological sections (at low magnification). (**A**) sagittal CT slice and (**B**) corresponding sagittal histological slice stained with van Kossa/McNeal stain. Both slices reveal a new bone formation on the side of the ilium, resulting in a depression in the subchondral bone of the sacrum. (**C**) sagittal CT section and (**D**) corresponding histological section stained with Giemsa stain. Multiple indentations are visible in the subchondral bone of the sacrum. (**E**) sagittal CT section and (**F**) corresponding histological section stained with Giemsa stain. Similar indentations are observed in the subchondral bone of the sacrum. (**G**) sagittal CT section and (**H**) corresponding histological section stained with Giemsa stain. Buttressing of the subchondral bone is evident on the sides of both the ilium (I) and the sacrum (S).

The histological grades of the SIJ strongly and significantly correlated with the overall CT grade (*p* = 0.011, correlation coefficient r_s_: 0.731, *n* = 11) (Figure 4A–F) and with the ultrasonographic grade (*p* = 0.024, correlation coefficient r_s_: 0.670, *n* = 11). This indicates that if horses exhibit alterations in the SIJ on CT and/or ultrasonography, they are likely to exhibit corresponding degenerative changes histologically (Figure 4A–F).

## 4. Discussion

This study aimed at describing, grading, and associating transrectal imaging and histological findings of SIJs of Warmblood horse cadavers with the hypothesis that SIJ changes are common in Warmblood horses. This assumption was correct because, although the degree ranged from mild to moderate, all equine SIJs showed abnormal CT findings, and 92% showed abnormal ultrasonographic findings. The CT characteristics of degenerative joint disease previously reported in humans [30] and dogs [18] were also detectable in our study population. This high prevalence of abnormal CT findings is consistent with the human literature, where degenerative changes of the SIJ in CT of asymptomatic humans are as frequent as the changes seen in patients with lower back pain [17]. In our study, the prevalence of the ultrasonographic findings was also high (92%). This is in line with the high prevalence of CT findings. A study evaluating 296 horses with a positive response to local infiltration of anesthetic solution in the SIJ region and abnormal radiopharmaceutical uptake on scintigraphy reported a substantially lower prevalence of ultrasonographic abnormalities within the SIJ (32%) [2]. A possible explanation for this discrepancy is that the ultrasonographic evaluation criteria were different. In Barstow et al., only SIJ modeling was assessed as a single abnormal joint-related criterion. In our study, multiple SIJ criteria were included, which may have resulted in stricter analysis and a larger number of joints being classified as having findings.

In horses, a high prevalence of macroscopic SIJ changes was observed in both healthy individuals and clinically affected horses based on pain localized to the SIJ using diagnostic anesthesia [3,23,28,31]. This indicates that adaptative modeling of the SIJ is likely depending on a horse’s conformation, posture, and use. Differentiating between normal adaptative changes and clinically relevant changes remains difficult. In human medicine, age-dependent clinically relevant imaging findings were only determined through a comparison of changes observed in clinically affected patients with sacroiliitis and those in asymptomatic individuals [30,32,33]. One study did not show any differences in gross-pathological changes in clinically symptomatic horses localized to the SIJ and control horses [28]. However, future studies comparing the diagnostic imaging findings in asymptomatic and symptomatic horses are essential to elucidate the clinical relevance of these findings. A close correlation of the diagnostic imaging findings with the results of the orthopedic examination as well as local anesthesia of the SIJ is imperative.

The alterations identified through ultrasonography and CT demonstrated a moderate, albeit non-significant, correlation. Thus, SIJ ultrasonographic findings are not directly representative of CT changes, and vice versa. Therefore, the second hypothesis, suggesting a strong correlation between ultrasound and CT findings, could not be accepted. A possible reason for this discrepancy is that the evaluation criteria of the modalities were not identical. A recent CT study of clinical cases also found that suspected lesions in the lumbosacroiliac region identified by ultrasound could not be confirmed by CT [34]. However, the observed weak positive correlation between CT and ultrasonographic findings could suggest a possible trend that might become significant with a larger sample size.

No significant differences were observed in the overall degree of CT changes between the caudal third of the SIJ and the overall degree of changes in the entire joint. However, consistent with previous reports [23,31], the most severe CT changes were localized in the caudal third of the SIJ. Therefore, the sole examination of the caudal third of the joint by transrectal ultrasonography should depict the most severe joint pathology in nearly half of the horses (44%). In cases refractory to treatment or exhibiting unusual clinical signs, both modalities should be used in a complementary fashion. Furthermore, pain from structures adjacent to the sacroiliac joint should be considered as a differential diagnosis. As local anesthetic solution may diffuse beyond the joint, sacroiliac analgesia is not necessarily specific, and surrounding regions should be carefully evaluated [35].

The buttressing changes observed in our study have previously been reported in the literature [3,31,34]. In 1999, Haussler et al. described periarticular new bone formation in racehorse specimens in which no clinical signs of SIJ pathology had been recognized, consistent with the buttress-type lesions observed in the present study [31]. In contrast, in horses with suspected SIJ disease, the joint surface is enlarged due to bony outgrowths along the margins, which may resemble the same lesion type [3,31]. Consequently, it remains unclear whether this type of finding has any clinical relevance.

Former studies describe that the equine articular surface of the sacrum is covered by hyaline cartilage and the auricular surface of the ilium by fibrocartilage [4,23,36]. This study shows contradictory results: hyaline cartilage was not only present in the articular surface of the sacrum but also in the central aspect of the articular surface of the ilium. Thus, this work confirms the presence of fibrocartilage and hyaline cartilage on both articular surfaces of the SIJ. Chondrocyte group formation with and without hypertrophy, the presence of pyknotic chondrocytes as well as a vascular perforation of the tide mark were histologically the most prevalent findings within the articular cartilage. Histological signs of joint degeneration within the SIJ were previously reported to occur with high prevalence in the articular cartilage of horses older than four years [36].

In human medicine, the presence of hypertrophic chondrocytes in cartilage without endochondral ossification is considered a definitive sign of osteoarthritis, as the shift of chondrocytes to a hypertrophic phenotype induces a series of events leading to articular cartilage degeneration [37,38]. In horses, endochondral ossification in the SIJ is complete by the age of four years [36]. All horses included in this study were older than four years. Consequently, the presence of hypertrophic chondrocytes in these horses can be considered a sign indicative of articular cartilage degeneration in the context of osteoarthritis. Whether these degenerative changes have current or future clinical relevance remains unclear. Currently, no imaging modality enables the direct evaluation of articular cartilage at the SIJ. However, this study demonstrated that when CT and/or ultrasonography reveal alterations in the SIJ, the articular cartilage also exhibits histological signs of joint degeneration. Thus, the third hypothesis of the study, proposing that horses with findings in the imaging procedures also show histological signs of joint degeneration, can be confirmed. Nevertheless, when the histological changes in the joints were considered collectively in the form of a total score, they were mostly categorized as mild. This may suggest that the frequently observed histological findings are not necessarily degenerative changes associated with osteoarthritis, but rather adaptative changes of the bone and cartilage to facilitate the intensive transmission of forces from the hindlimbs to the spine. In this context, cartilage cell hypertrophy could represent a response to increased cellular abrasion on the joint surface, which, during loading, must withstand high pressure on the small, closely opposing cartilage surfaces.

Most of the histological changes in the articular cartilage and subchondral bone were significantly more prevalent on the sacral articular surface compared to the iliac articular surface.

Previous post-mortem studies in the horse revealed a high prevalence of macroscopic visible degenerative changes on both joint surfaces (100% in the sacrum and 72% in the ilium) [31,39,40]. These changes were not only more frequent but also exhibited greater severity on the sacral joint surface [31]. In contrast, osteophytes identified within the SIJ on post-mortem CT images appeared to be symmetrically distributed across both joint surfaces [21]. The observed differences in the extent of degenerative changes between the iliac and sacral joint surfaces may be explained by variations in the mechanical forces acting on each bone. Haussler et al. reported subtle, independent motion patterns of the ilium and sacrum, suggesting that these structures may not experience identical mechanical loading [41]. It has been postulated that shear forces predominantly influence the SIJ, as opposed to compressive forces, resulting in diminished synovial turnover and, consequently, impaired nourishment of the articular cartilage [23]. This impaired cartilage nutrition could also contribute to the high prevalence of degenerative changes observed within the equine sacroiliac joint.

The most significant limitation of the study was the absence of a thorough lameness examination by an experienced clinician familiar with the clinical signs indicative of SIJ disease conducted before the horse’s death. Theoretically, horses with early stages of SIJ disease with only mild clinical signs which may have been missed by the owners could have been included in the study. All horses were used at their original riding level before being presented to the clinic for euthanasia/slaughter, and they did not show any reported evidence of clinical signs typical of SIJ disease. Nevertheless, it should be noted that a previous study demonstrated that approximately 50% of horses considered riding sound by the owner exhibited lameness when evaluated at the trot [42]. However, no clinical signs attributable to early SIJ disease have been described to date, which is why these cases could also have been missed during an orthopedic examination.

Due to the absence of horses without CT-detectable lesions, test characteristics such as specificity and negative predictive value of ultrasonography relative to CT could not be calculated. This represents a limitation of the study.

For ethical reasons, the imaging modalities were only carried out postmortem. This inevitably led to minor gas artifacts within the vasculature overlying the SIJ on ultrasonography. This was most pronounced when horses were slaughtered due to gas entering the common carotid artery when it was severed. The CT quality was not affected by the gas artifacts. Furthermore, CT scans of living horses under general anesthesia may demonstrate reduced spatial resolution compared to cadaveric studies due to the increased anatomical mass [22] and motion artifacts. Similarly, ultrasound examination in live horses can be more challenging than post-mortem imaging because of muscle contractions, large blood vessels, etc. Therefore, the results of this study should not be directly extrapolated to clinical cases.

## 5. Conclusions

SIJ findings in CT, ultrasonography, and histology are very common in adult Warmblood horses without owner-perceived clinical signs indicating SIJ disease. These may represent adaptative to early maladaptive joint modeling. Future studies investigating the clinical relevance of these findings and/or the pattern of changes in horses presenting with clinical symptoms represent the next step in advancing this research. The findings of the ultrasonographic and CT examinations showed non-significant correlations, which is likely due to the criteria evaluated. The overall CT findings in the caudal third of the SIJ did not significantly differ from those present in the entire joint. Additionally, the most severe CT lesions were most frequently located in the caudal third, further supporting the diagnostic relevance of this region, assessable by ultrasonography. In horses exhibiting alterations in the SIJ detected by CT and/or ultrasonography, corresponding degenerative changes of the articular cartilage by histology can be expected.

## Figures and Tables

**Table 1:** Gradings (0–3) of the transrectal ultrasonographic findings of the sacroiliac joint of the examined cadavers according to Tallaj et al. [10]. The distinction between osteophytes and enthesophytes was made according to their anatomical location: bony projections along the joint margin were classified as osteophytes, while those located at the insertion site of the ventral sacroiliac ligament—further from the joint line—were considered enthesophytes. Fibrotic changes in the ventral sacroiliac ligament were characterized by diffuse hyperechoic fiber patterns without acoustic shadowing. Ligamentous atrophy was defined by thinning/narrowing of the ligament. Modeling of the bones was defined as an irregular bone contour.

Grade	Articular Margins	Modeling	Enthesophyte Formation	Ventral Sacroiliac Ligament	Joint Effusion
0	Normal articular margins	No change of shape	No enthesophytes present	Normal echogenicity of ventral sacroiliac ligament	No joint effusion
1	Mild irregular articular joint margins	Mild modeling	Mild enthesophyte formation	Heterogenous echogenicity of ventral sacroiliac ligament	Suspected or mild effusion
2	Moderate irregular articular joint margins	Moderate modeling	Moderate enthesophyte formation	Fibrosis and/or atrophy of the ventral sacroiliac ligament	Moderate joint effusion
3	Severely irregular articular joint margins	Severe modeling	Severe enthesophyte formation	Severe fibrosis and/or atrophy of the ventral sacroiliac ligament	Severe joint effusion

**Table 2:** Gradings (0–3) of the computed tomographic sacroiliac joint findings adapted from Wise et al. [20]. Intra-articular bone spurs were defined as bony proliferations extending into the joint space, while ankylosis referred to bone proliferation bridging the SIJ with focal loss of joint space. Subchondral erosions were described as focal concavities with ill-defined margins along the articular surface. Bone cysts were characterized as oval, hypoattenuating defects with smooth margins within the subchondral bone. Subchondral sclerosis was defined as an area of increased attenuation within the subchondral region. Bone modeling refers to an irregular contour or shape of the sacral or iliac joint surface.

Grade	Osteophyte Formation	Periarticular Modeling (Enthesophyte Formation)	Subchondral Bone Lesions	Subchondral Bone Sclerosis	Sacrum/Ilium Bone Modeling
0	Normal	Normal	Normal	Normal	Normal
1	Periarticular osteophytes	Mild enthesophyte formation	Mild erosion and/or one small cyst	Mild/focal sclerosis	Mild modeling
2	Intra-articular bone spurs	Moderate enthesophyte formation	Moderate erosion and/or multiple cysts	Moderate/multifocal sclerosis	Moderate modeling
3	Intra-articular ankylosis	Severe enthesophyte formation and/or ankylosis	Severe erosion and/or >3 cysts	Severe/diffuse sclerosis	Severe modeling

**Table 3:** Microscopic grading system used for evaluation of articular cartilage, subchondral bone, and joint capsule findings in the sacroiliac joint, adapted from Mcllwraith et al. [29].

Histological Feature	Grade	Description
**Articular cartilage**		
Hypertrophic chondrocytes without group formation	0	No hypertrophic chondrocyte
	1	Isolated hypertrophic chondrocytes
Chondrocyte group formation without hypertrophy	0	No groups (>2 cells) without hypertrophy
	1	Groups (>2 cells) without hypertrophy
Chondrocyte group formation with hypertrophy	0	No groups (>2 cells) with hypertrophy
	1	Groups (>2 cells) with hypertrophy
Hypertrophic chondrocytes adjacent to fissures	0	No hypertrophic chondrocytes adjacent to fissures of the articular cartilage
	1	Hypertrophic chondrocytes adjacent to fissures of the articular cartilage
Pyknotic chondrocytes	0	≤1 pyknotic chondrocyte near the articular surface (20× magnification)
	1	>1 pyknotic chondrocyte near the articular surface or in the basal layer
Indentations in the cartilage without disturbance of the cartilage alignment	0	No indentation; cartilage alignment intact
	1	Indentation present; cartilage alignment intact
Indentations in the cartilage with disturbance of the cartilage alignment	0	No indentation
	1	Indentation present with disturbed cartilage alignment
Tidemark	0	Tidemark intact
	1	Tidemark penetrated by vessels
**Subchondral bone**		
Subchondral bone regularity	0	Regular subchondral bone
	1	Irregular subchondral bone
Subchondral bone thinning	0	Subjectively normal subchondral bone thickness
	1	Subjectively reduced subchondral bone thickness
Subchondral bone thickening/sclerosis	0	Subjectively normal subchondral bone thickness
	1	Subjectively increased subchondral bone thickness
Subchondral bone lesions	0	No lesion in the area of the subchondral bone
	1	Presence of subchondral bone cysts, fissures, clefts or several of these within the subchondral bone
**Joint capsule**		
Cellularity of the joint capsule	0	Normal cellularity
	1	Increased cellularity

**Table 4:** The prevalence of the most observed histological signs indicative of degenerative changes is presented. The analysis was performed on 104 histological sections from 11 randomly chosen sacroiliac joints. The most frequent findings included cellular changes, such as group formation of chondrocytes (defined as clusters of more than two cells) with and without hypertrophy, presence of pyknotic chondrocytes, and hypertrophy without group formation, as well as structural alterations including cartilage indentations with or without disruption of cell alignment, vascular disruption of the tidemark, and subchondral bone changes, such as sclerosis, irregularities, and lesions (e.g., cysts, clefts). Findings that were significantly more prevalent on the sacral joint surface are indicated by an asterisk (*), with corresponding *p*-values provided in parentheses.

Histological Finding	Iliac Joint Surface (% of Occurrence in 104 Histological Slides)	Sacral Joint Surface (% of Occurrence in 104 Histological Slides)
Group formation (>2 chondrocytes) without hypertrophy	44.3%	* 61.3% (*p* = 0.015)
Group formation (>2 chondrocytes) with hypertrophy	17%	* 50% (*p* < 0.001)
Pyknotic chondrocytes	26.4%	* 42.5% (*p* = 0.016)
Vascular disruption of tidemark	17.9%	* 30.2% (*p* = 0.25)
Hypertrophy without group formation	7.5%	* 21.7% (*p* = 0.004)
Cartilage indentations with disruption of cell alignment	21.7%	26.4%
Cartilage indentation without disruption of cell alignment	10.4%	11.3%
Sclerosis of subchondral bone	15.1%	* 40.6% (*p* < 0.001)
Irregular subchondral bone	21.7%	33%
Subchondral bone lesions (cysts, clefts…)	26.4%	31.1%

## Data Availability

The data that support the findings of this study are available from the corresponding author upon reasonable request.

## Abbreviations

The following abbreviations are used in this manuscript:

CT	Computed Tomography
ECVDI	European College of Veterinary Diagnostic Imaging
ECVS	European College of Veterinary Surgery
SIJ	Sacroiliac Joint
US	Ultrasonography
